# Oral Candidiasis in Adult and Pediatric Patients with COVID-19

**DOI:** 10.3390/biomedicines11030846

**Published:** 2023-03-10

**Authors:** Massimo Pisano, Antonio Romano, Maria Pia Di Palo, Adone Baroni, Rosario Serpico, Maria Contaldo

**Affiliations:** 1 Department of Medicine, Surgery and Dentistry, University of Salerno, 84084 Salerno, Italy; 2 Multidisciplinary Department of Medical-Surgical and Odontostomatological Specialties, University of Campania “Luigi Vanvitelli”, 80138 Naples, Italy; 3 Unit of Dermatology, Department of Mental Health and Physics and Preventive Medicine, University of Campania “Luigi Vanvitelli”, 80138 Naples, Italy

**Keywords:** oral candidiasis, COVID-19, SARS-CoV-2, *Candida albicans*, fungi, infection

## Abstract

Oral Candidiasis (OC) is an opportunistic fungal infection of the oral cavity, frequently reported under local and systemic predisposing circumstances. While the recurrence of OC HIV-infected subjects has been well described and reported, the association between oral candidiasis and the SARS-CoV-2 infection is a recent finding that still is worthy of further study. The present paper focuses on this novel association, reporting the incidence and prevalence of OC occurring during and after COVID-19 and the possible etiopathogenic mechanisms underlying the onset of OC in COVID-19 subjects. The work found that the immune inflammatory hypo reactions and immunosuppression found in children and adults with COVID-19 could favor the proliferation colonization of *Candida *species and the following infection. At the same time, poor oral hygiene and iatrogenic causes seem to be the main risk factors.

## 1. Introduction

Oral candidiasis (OC) is an opportunistic infection affecting the oral mucosa and arising from an increase in virulence of otherwise saprophytic yeasts of *Candida* species under predisposing systemic and local conditions [1]. Among them, xerostomia and unsanitized dental prostheses are the main predisposing local factors, thus exposing the elderly and denture carriers to a higher risk of OC [2,3,4,5]. The most associated extra-oral conditions are related to age (mainly children and elderly) [6], bad habits such as smoking and poor oral hygiene [7,8], syndromic or genetic disorders [9,10,11] iatrogenic causes (broad-spectrum antibiotic therapy and the use of immunosuppressors and/or steroids) [12], and chronic and systemic diseases, such as diabetes mellitus and immunodeficiencies [13,14].

While the onset of OC in the course of HIV infection has historically rooted-although the changes in epidemiology and prognostic value related to the antiretroviral therapy (ART) era [15,16,17,18], the association between oral candidiasis and the novel severe acute respiratory syndrome coronavirus 2 (SARS-CoV-2) infection is a recent finding that still is worthy of further study. The present paper aims to focus on this topic, reporting the incidence and prevalence of oral candidiasis COVID-19-associated and the possible etiopathogenic mechanisms underlying this association.

## 2. Prevalence of Oral Candidiasis in Adult and Pediatric Patients with COVID-19

The oral cavity is an entry route for the human body that hosts a complex and dynamic microbiota, but not pathogenic in a healthy state [19]. However, bacteria, archaea, fungi, or viruses can cause tissue damage when the balance of microorganisms is perturbed [19]. The oral also microbiota comprises a minority of microorganisms of the kingdom of Fungi [19,20]. Oral fungal infections include mucormycosis, blastomycosis, coccidioidomycosis, aspergillosis, cryptococcosis, and histoplasmosis [19,20], but candidiasis is the most common, sustained by different *Candida* species, mainly *C. albicans*.

*Candida albicans* is an oral fungal commensal present in almost 40–65% of healthy adults [21] but capable of causing opportunistic infections, significantly when the immune system is compromised [22]. Several cases of oral candidiasis in SARS-CoV-2-positive subjects have been reported in the literature [21,23,24,25,26]. In subjects with COVID-19 and oropharyngeal candidiasis, *Candida albicans* was isolated more frequently (70.7%), followed by *C. glabrata* (10.7%), *C. dubliniensis* (9.2%), *C. parapsilosis* (4.6%), *C. tropicalis* (3%), and *C. krusei* (1.5%) [21]. 

A recent systematic review evaluated the prevalence of oral manifestations in 4925 adults (>18 years) positive for SARS-CoV-2, and oral candidiasis was found in 10.74% of cases [24]. A similar study included 35 pediatric subjects (<18 years) recording oral candidiasis in 11.63% of cases [23]. A previous study [27] from Iran reported, among 1059 with COVID-19, a lower percentage of oral candidiasis, equal to 5%. Probably, the percentage was underestimated because all patients who had developed Acute Respiratory Distress Syndrome (ARDS) were excluded from this study.

COVID-19 treatment may require antibiotics or corticosteroids, whose usage is frequently associated with the onset of oral candidiasis. Babamahmoodi et al. [28] reported that patients with COVID-19 and under corticosteroid or antibiotics therapy showed more candidiasis than patients SARS-CoV-2 positive, not under pharmaceutical treatment. Salehi et al. reported similar conclusions, establishing that 92.5% of the oropharyngeal candidiasis in COVID-19 patients was associated with the usage of broad-spectrum antibiotics [27]. The mechanism by which antibiotics and corticosteroids promote opportunistic *Candida* infection will be explained more fully in Section 3.5.

Elderly patients with chronic medical diseases, such as arterial hypertension, diabetes, and cardiovascular diseases, share a higher risk of developing both severe COVID-19 [25,29,30] and oropharyngeal candidiasis [25,27]. In detail, subjects SARS-CoV-2 positive older than 50 years were at higher risk of developing oral candidiasis, and the predisposing condition most commonly associated was lymphocytopenia (71%) [27].

Denture wearers are generally more prone to suffer recurrent oral candidiasis, also defined in this case as denture stomatitis. Regarding the denture wearers with COVID-19, it has been reported that 60% of them also had denture stomatitis, mainly sustained by *C. albicans *strains [25].

## 3. Possible Mechanisms Underlying Oral Candidiasis in Adult and Pediatric Patients with COVID-19

### 3.1. The Role of Immune-Inflammatory Dysregulation for Candida Co-Infections in COVID-19 Patients

A putative pathogenetic mechanism for *Candida* co-infections in SARS-CoV-2 positive patients could be linked to immune-inflammatory hypo-reactions [21]. Immunosuppression is often associated with SARS-CoV-2 infection [29] with decreased CD4+ and CD8+ T cells [31]. This immune hypo-dysregulation caused by COVID-19 could favor the opportunistic infection by *Candida*, similar to what happens in HIV-positive subjects with decreased levels of CD4+ cell count [32]. In fact, Shekatkar et al. [32] reported that the prevalence of oral candidiasis in HIV seropositive was significantly reduced after the Highly Active Antiretroviral Therapy (HAART) regimen. This decreased prevalence may be associated with the improvement of the immune system.

Furthermore, in COVID-19 patients, in addition to lymphopenia, T lymphocytes are polarized toward the spike protein [33]. This state could lead to opportunistic *Candida* infections, similar to Herpesvirus Simples-1 or Varicella Zoster infections, which are latent in neuronal ganglia and can reactivate in SARS-CoV-2 positive subjects, also inducting oral manifestations [34]. Last, adults are subject to a natural process of immunosenescence [35] and consequent further depletion of T lymphocytes. Thus opportunistic *Candida* infection due to immune hypo reaction may be more common in adults. In addition, other authors have reported that several pathogenic mechanisms occurring during COVID-19, such as immunosuppression and pro-inflammatory cytokines cascade, associated with the reduced fungicidal activity of neutrophils may cause direct damage to the oral mucosa and contribute to streamlining the *Candida* infections [36].

### 3.2. The Role of Direct Viral Cytopathic Effect of SARS-CoV-2 for Candida Co-Infections in COVID-19 Patients

COVID-19 patients showed lower levels of zinc, vitamins A, B_6_, C, D, and E [37]. One of the predisposing factors of oral candidiasis is nutritional deficiency, such as vitamin A, B_6_, and iron [38]. Paillaud et al. [39] suggested the hypothesis that these vitamin deficits alter the oral mucosa integrity, thus leading to the pathogenesis of oral fungal colonization. Furthermore, SARS-CoV-2 could also induce a direct viral cytopathic effect [34] because the virus can penetrate the oral epithelial cells through the angiotensin-converting enzyme 2 (ACE2) receptors expressed by oral epithelium and ductal cells of salivary glands causing the cells apoptosis. The salivary glands’ invasion by SARS-CoV-2 leads to xerostomia [40], another predisposing factor of oral candidiasis because of the reduction of histatin, calprotectin, and antifungal proteins as lysozyme or lactoferrin [21,38,41]. The salivary flow is furtherly reduced in prosthetic wearers and patients in ICU with mechanical ventilation [25]. Saliva plays an essential role in the equilibrium of the oral microbiota [42]. Low salivary pH and oral clearance caused by COVID-19 create alterations in the biofilm composition due to the reduction of essential proteins like mucins compromising their role of blocking microorganisms’ adherence to oral surfaces [42]. This perturbation increases the risk of gingivitis, caries, and fungal infections.

### 3.3. The Role of Lifestyles and Oral Care Changing for Candida Co-Infections in COVID-19 Patients

During COVID-19, physical distancing and self-isolation have widely affected lifestyles, particularly eating habits and everyday behaviors such as oral care routines [43,44,45,46].

In detail, a reduction of oral hygiene care was noted among adolescents during the self-isolation, when only 39.1% brushed their teeth more than three times daily, compared with 47.8% before it; on the other hand, 52.2% affirmed that they brushed their teeth less than three times daily before the physical distancing, compared with 60.9% during it [45,46]. Similarly, SARS-CoV-2-positive patients may encounter an improvement or a worsening of oral hygiene during self-isolation. When there is a thicker oral biofilm, *Candida albicans* increase its expression of some hyphal genes, like *Hwp1*, while its production is lower in thinner biofilms [47]. These genes help the transition from commensal to pathogenic *Candida* [48].

In addition, some studies have reported worsening eating habits [23,45]. Jia et al. [44] assessed the frequency of food intake and the relative amount of carbohydrates and snacks. Many Authors [44,45,46] valued the kind of foods chosen, including the number of fresh foods such as fruits, vegetables, and fish. It was reported that people during isolation consumed more canned foods and sweets in high frequency compared to healthy foods [46]. Increased glucose levels in saliva may promote host susceptibility to oral infections, such as candidiasis [49]. Glucose regulates the expression of aspartyl proteinases (SAP) 4, 5, and 6, which are associated with the development of hyphae favoring the infection and persistence in oral tissues of *Candida albicans* [49].

### 3.4. The Role of ICU and Hospitalization for Candida Co-Infections in COVID-19 Patients

Most cases of *Candida* infection in COVID-19 patients occur in subjects admitted at the intensive care units (ICUs), meanly after a week from the recovery [50,51], being responsible for a two-fold mortality risk compared to patients with candidemia without COVID-19 [36,50,52].

*Candida* was among the most common microorganisms detected in 6% to 10% of patients with COVID-19 treated in the intensive care unit (ICU) [25]. Indeed, the Centre for Disease Control COVID-19 Response Team reported that 33% of subjects between 18 to 64 years old were hospitalized compared to 20% of pediatric SARS-CoV-2 positive patients [53]. In detail, mechanical ventilation was necessary in about 8.3% of adult hospitalized COVID-19 cases [21] and 2.1% of pediatric ones [54]. In fact, according to Nambiar et al. [21], patients with COVID-19 in the ICU have a 10-fold higher risk of developing bacterial or fungal secondary infections than secondary viral infections. In these patients, *Candida* was found in more than 78% of respiratory specimens [25]. The reason could be that pathogens of oral biofilm may be aspirated by mechanical intubation, as claimed by Jerônimo et al. [25]. In their study, the same microorganisms were found in tracheal aspirate and oral biofilm in 59.37% of intubated patients [25]. In these cases, there was a risk of candidemia, ventilator-associated pneumonia (VAP) in 42.1% of cases [25], aspirated pneumonia in 5.2% [25], and death in 70% [22]. On the other hand, for patients not in ICU, the risk of death from candidemia is lowered between 19% and 40% [22].

Furthermore, patients under mechanical ventilation also develop xerostomia, and the oral biofilm control worsens. These factors, as previously shown, favor opportunistic *Candida albicans* infection.

### 3.5. The Role of Drugs for Candida Co-Infections in COVID-19 Patients

The medicinal products commonly used to treat COVID-19 include antivirals, antibiotics, anthelmintics, anti-inflammatory drugs (corticosteroids, acetylsalicylic acid, indomethacin), intravenous immunoglobulins, interferon, monoclonal antibodies, anticoagulants, vitamins C, D and zinc [54].

As anticipated above, over 90% of the oropharyngeal candidiasis in COVID-19 patients is strictly related to broad-spectrum antibiotics usage [29], especially when empirically prescribed [55].

*Candida* colonization was also significantly associated with the use of steroids [40], capable of inducing a breakdown of the oral microbiome, as long-term and broad-spectrum antibiotics [56]. While in normal health conditions, the growth of *Candida* is prevented by other oral microbiota microorganisms [21], long-term treatment with antibiotics or corticosteroids favors oral candidiasis in the form of thrush or, more commonly, acute erythematous candidiasis [41,57].

Knight et al. [58] suggested that antibiotics may also suppress the production of anticandidal antibodies and phagocytosis or stimulate fungal growth by inhibiting salivary bacteria [57,58]. In addition, the glucose level in the saliva of patients who had taken antibiotics was measured and found to be increased to mean values of 7.41 mg/100 mL compared to 0.05 mg/100 mL in the control group [58].

Corticosteroids can be used for as short as 3–5 days in COVID-19 patients with progressive deterioration of oxygen saturation, high proinflammatory response, and rapid worsening on chest imaging [59], thus probably decreasing all-cause of death in hospitalized patients with COVID-19 [60]. Inhaled corticosteroids also reduce admission to hospital or death and speed up the resolution of symptoms in COVID-19 patients with mild symptoms [61]. However, the co-usage of systemic and inhaled corticosteroids is associated with an increased risk of oral candidiasis.

Corticosteroids probably favor opportunistic *Candida* infection due to their anti-inflammatory and immunosuppressive effect [62]. In addition, patients on steroid treatment have more glucose in their saliva than controls, which could favor *Candida*’s growth, proliferation, and adhesion to oral mucosa cells [58]. Higher glucose levels in saliva also decrease pH levels, favoring *Candida*’s secretion of aspartyl proteinases and phospholipase, enzymes that increase the fungus’s pathogenicity [62].

Furthermore, dexamethasone is also internalized by *Candida* cells and can promote the adhesion of the fungus to oral mucosa cells by interacting directly with surface receptors [63]. Kuna et al. [64] reported that oral candidiasis occurred in 10–30% of patients using inhaled corticosteroids for long times, and according to Doğan et al. [65], 37.6% of children who use inhaled corticosteroids for asthmatic problems develop oral candidiasis. Ciclesonide is converted to its active metabolite (desisobutyryl-ciclesonide) in the lungs and has a strong antiviral effect in the alveoli [20]. The deposition and activation of ciclesonide are low in the oropharynx, and probably because of this, the incidence of oral candidiasis from ciclesonide is lower than that due to other inhaled steroids [20]. However, cases of oral candidiasis from ciclesonide in SARS-CoV-2-positive patients have also been reported [20].

## 4. Discussion

**Figure 1:**
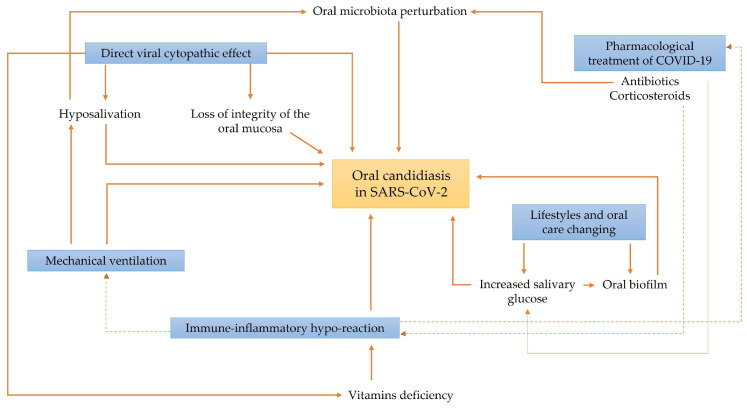
The risk factors in COVID-19 subjects favoring the onset of oral candidiasis.

The present work aimed to discuss the recent evidence from the literature about the association between oral candidiasis (OC) and the novel SARS-CoV-2 infection by reporting the epidemiology in children and adults of “COVID-19 associated candidiasis”, CAC, focusing on the oral manifestations [66], and the possible etiopathogenic mechanisms underlying this association, relating them with the potential risk factors, summarized in Figure 1.

In summary, the reports from the literature revealed a higher risk of contracting oral candidiasis during and after COVID-19, mainly in those patients admitted to the ICU and under mechanical ventilatory support, who have significantly reduced salivary flow, use broad-spectrum antibiotics to treat VAP, and cannot carry out routine oral hygiene practices, being intubated.

Data suggest the main risk factors for the onset of oral candidiasis in COVID-19 patients are attributable to direct and indirect effects of the SARS-CoV2 (immunity dysregulation and viral cytopathic effects on ductal epithelial cells the salivary glands [40] expressing high levels of ACE2 [67], responsible for xerostomia) and drugs, essential to treat COVID-19 [40], mainly long-term antibiotics and steroids, which alter the oral microbiota and suppress the immune surveillance, thus favoring opportunistic fungal infections.

Furthermore, poor oral hygiene, secondary to changes in routine oral care and eating habits, reported during the lockdown period [46,68,69], as well as the increase in salivary glucose resulting from the usage of corticosteroids and some antibiotics [49,58], favor the establishment of an oral environment more conducive to the development of opportunistic infections, by favoring the dental biofilm growth and maturation, which promotes the adhesion of *Candida* species and reduce the production of antimicrobial proteins such as lysozyme and lactoferrin, thus impoverishing the antimicrobial power of saliva [21,57].

## 5. Conclusions

From the analysis of the literature, a high prevalence of oral candidiasis in young and adult patients suffering from COVID-19 emerged since an opportunistic infection as the OC is, is clearly advantaged by the local and systemic conditions established by SARS-CoV2 infection itself and the COVID-19 medical treatments and procedures.

However, it has emerged that the lymphopenia typical of COVID-19 is not the (only) cause of enhanced susceptibility to CAC and OC, but multiple etiopathogenic mechanisms interplay and favor the onset of OC after SARS-CoV2 reinfection [70].

In addition to the literature reported, scientific research still focuses on the interplay between oral candidiasis and SARS-CoV2 and studies specific therapeutic approaches directed toward CAC and OC/COVID-19. For example, Yang et al. considered the co-occurrence of OC and COVID-19 as a unified pathologic entity (OC/COVID-19) to treat with appropriate and specific drugs [71]. To discover the most effective drugs active toward OC/COVID-19, they search for and found nine hub genes with a high correlation between OC and COVID-19 and toward which the same drug can be equally effective. The genes found were primarily those involved in the IL-1 and TNF pathways, and, for four of them, five specific drugs -mainly IL-1 specific inhibitors and over twenty herbs from traditional Chinese medicine exist [71]. However, we are still far from their clinical application.

Now that we have passed the critical phase of the SARS-CoV2 pandemic and we are living with a virus towards which we have developed adequate vaccination and therapeutic strategies to reduce mortality, we are now facing a second phase during which COVID-19 is fortunately treatable and can deal with the health consequences that it determines.

We can focus and dedicate ourselves to addressing all those comorbidities, direct and indirect sequelae of COVID-19, which afflict the patient’s health, and which should not be underestimated, especially in fragile subjects.

Concerning oral health and the increased risk of oral candidiasis, it could be feasible to sensitize the doctors engaged in the wards of COVID-19 departments and especially dentists and physicians in the territory, to support oral health in COVID-19 patients by preventive measures to avoid or reduce the occurrence of opportunistic infections such as oral candidiasis, which can be the springboard for invasive fungal diseases.

Examples of such preventive interventions in the case of noncritical COVID-19 adults and children who do not require hospitalization and spend their stay at home in solitary confinement are [69,72,73]:

- food education and oral hygiene;
- reducing the use of antibiotics and corticosteroids only to cases strictly necessary;
- and oral health monitoring with periodic visits to the dentist.

Last, particular attention should also be paid to those hospitalized once the vital parameters have been stabilized, and those at greater risk, the elderly, the edentulous, those with multiple comorbidities, and all the fragile subjects, which are more prone to develop not only oral candidiasis but also invasive fungal infections.
